# Domain-specific life satisfaction among older adults with and without children: The role of intergenerational contact

**DOI:** 10.1371/journal.pone.0257048

**Published:** 2021-09-22

**Authors:** Friederike Doerwald, Birte Marie Albrecht, Imke Stalling, Karin Bammann

**Affiliations:** Working Group Epidemiology of Demographic Change, Institute for Public Health and Nursing Sciences (IPP), University of Bremen, Bremen, Germany; Prince Sattam Bin Abdulaziz University, College of Applied Medical Sciences, SAUDI ARABIA

## Abstract

**Background:**

Life satisfaction is associated with many important health outcomes among older adults and is an indicator of successful ageing. The present study aims to replicate earlier findings regarding relationships between satisfaction with various life domains and life satisfaction in older adults. The study furthermore explores how parental status is associated with satisfaction with different life domains and how two types of intergenerational contact (contact with own children; post-retirement work in childcare) relate to life satisfaction.

**Methods:**

Participants were 1978 older adults, aged 65–75 year (51.7% female), who live in Bremen and took part in the OUTDOOR ACTIVE study. 82.6% of the participants had one or more children. All participants completed a questionnaire, which among others comprised items assessing life satisfaction as well as satisfaction with six different life domains (satisfaction with living situation, financial situation, leisure time, health, family, neighbors and friends).

**Results:**

LS is significantly related to all of the investigated life domains, independent of sex and age. For the participants with children, life satisfaction had the highest association with satisfaction with family (β: 0.202; 95%CI: 0.170–0.235), followed by satisfaction with neighbors and friends (β: 0.151; 95%CI: 0.111–0.191), and health satisfaction (β: 0.148; 95%CI: 0.120–0.176). In comparison to that, participants without children had the highest association between life satisfaction and satisfaction with health (β: 0.193; 95%CI: 0.135–0.252), followed by satisfaction with family (β: 0.175; 95%CI: 0.114–0.236) and satisfaction with neighbors and friends (β: 0.154; 95%CI: 0.077–0.232). In participants with children, there was a non-significant negative association between life satisfaction and work in childcare (β: -0.031; 95%CI: -0.178–0.116), while life satisfaction was statistically significantly positively associated to work in childcare in participants without own children (β: 0.681; 95%CI: 0.075–1.288).

**Conclusions:**

The results suggest that the domain-specific approach to life satisfaction can elucidate differences in the correlates of life satisfaction and well-being between older adults with and without children. They further suggest that the benefits of working with children for life satisfaction may be more pronounced in older adults without children than older adults with children.

## Introduction

Life satisfaction (LS) pertains to people’s cognitive evaluation of their overall life quality [1]. Together with an affective component (i.e. positive and negative affect), it constitutes the concept of subjective well-being [2]. A number of longitudinal and twin studies have demonstrated the beneficial effects of LS for health outcomes [3], such as better physical health [4], lower risk for depression [5], and lower unnatural-cause as well as unintentional injury mortality [6, 7]. LS is also an indicator of successful ageing [8, 9] and a body of research has investigated LS in the population of older adults. Similar to findings from studies with age-heterogeneous samples, older adults’ LS is associated with health outcomes, such as self-reported health [10], depressive symptoms [11], and mortality [12], highlighting the importance to identify potential predictors and understand the trajectories of LS among older adults.

Despite age-related cognitive and physical declines, longitudinal studies suggest that subjective well-being in general, including LS, does not decrease in old adulthood [13]. Instead, research has repeatedly found that LS follows a U-shape across the adult life span, observing the lowest LS among adults between 30 and 50 years [14]. Note that some research indicates another drop in LS in old age, which might be attributed to reductions in perceived health [15]. Previous research has also identified a number of potential determinants of older adults’ LS, including demographic variables, for instance gender [16], psychosocial variables, such as personality [17] and social relationships [18, 19], and health-related variables, as for example self-reported health and health-related behavior [10, 20–22].

While most studies have focused on overall LS of older adults, another approach is to differentiate among satisfaction with various life domains [23]. The latter takes into account that people may overall be satisfied with their life but still experience dissatisfaction in certain domains and that some life domains may affect overall LS stronger than others [24]. Approaching LS as a multidimensional construct can hence shed light on the potential sources of overall LS [25]. Meta-analytic evidence suggests that satisfaction with key life domains (job, marriage, health, and social life) is related to LS, with effect sizes being moderate [26]. The present study approached LS as a multi-dimensional construct, assessing satisfaction in six key life domains (i.e. satisfaction with living situation, financial situation, leisure time, health, family, neighbors and friends) that were derived from a nationally representative study [27] and that largely overlap with domains in life that have repeatedly been identified as relevant to LS (e.g. [23, 28]).

One particularly important predictor of LS in older adults is satisfaction with social relationships, such as friends and family [10]. In fact, family relations might be even more important for LS than relationships with friends. Accordingly, research has shown that family support is a stronger contributor to older adults’ LS than support from friends [19]. One major source of family support are *intergenerational family relations*, including relationships with grown children or grandchildren. Thus, it is conceivable that intergenerational contact within families is positively related to LS through increased levels of support gained from these relationships. However, older adults’ amount of intergenerational contact within the family may differ, depending on the amount of living children [29]. Childless older adults appear to have less intergenerational family contact and may seek other ways to interact with younger people [30]. One way to compensate limited intergenerational family contact could be through engaging in either paid or unpaid work with children.

The goal of the present study is to first replicate earlier findings regarding the contribution of satisfaction with different domains in life to overall LS in a sample of older adults, aged 65–75, who participated in the OUTDOOR ACTIVE study. Considering the importance of having intergenerational family relations, which are affected by parental status [30], for well-being (e.g., [31]), we furthermore explore differences between older parents and childless older adults regarding associations between domains of LS and LS. We also examine how doing work in childcare relates to LS of older adults with and without children.

## Methods

### Study design and population

OUTDOOR ACTIVE is part of AEQUIPA, a prevention research network investigating physical activity as a key determinant of healthy ageing [32]. The aims of the OUTDOOR ACTIVE study are to explore drivers and barriers for being physically active, and to develop and implement a community-based outdoor physical activity promotion program for older adults [33]. The study comprises a pilot study (February 2015 –January 2018) and a cluster-randomized controlled trial (c-RCT) (February 2018 –January 2021; German Clinical Trials Register: Deutsches Register Klinische Studien DRKS00015117), both consisting of a baseline and follow-up survey using 1) a self-administered paper-pencil questionnaire, including intrapersonal, interpersonal, and environmental determinants of physical activity, 2) a brief physical examination, including anthropometry and blood pressure, followed by a fitness test (modified Senior Fitness Test [34] and handgrip strength test), and 3) a seven-day accelerometer measurement. Eligibility criteria for both study parts were 1) being between 65 and 75 years old, 2) residing in defined sub-districts of Bremen, Germany (pilot study: Arbergen, Hastedt, Hemelingen, Mahndorf, Sebaldsbrück; c-RCT: Blumenthal, Burg-Grambke, Gete, Lehe, Lehesterdeich, Neustadt, Ohlenhof, Ostertor), and 3) being non-institutionalized. Address data were provided by the registry office of Bremen. Eligible individuals initially received a letter and were later contacted by phone, if the numbers could be obtained.

In total, 11,079 individuals were registered at a private household in the included sub-districts of the pilot study and the c-RCT. Of those, 125 were deceased and 450 had moved out of the study region at the time of the survey, and were thus not eligible to the surveys. Exclusion criteria were acute disease (e.g. infections; n = 461) or not being able to communicate in German, English or Turkish (n = 77). 9,966 confirmed eligible individuals remained, of which 3,425 were never reached and 4,247 refused to participate. 151 individuals of the sub-district Lehesterdeich were never contacted, since the survey period for that region ended and the sample size had already been exceeded at that point. Ultimately, 2,143 participated in at least one part of the pilot study or the c-RCT. Of those, 1,978 participants filled out the questionnaire, including outcome and exposition variables, and were therefore included in the present study.

All participants provided written informed consent and both study parts were approved by the ethical committee of the University of Bremen.

### Measures

All variables were assessed using a self-administered questionnaire. The questionnaire was available in German and Turkish, help for filling in the questionnaire was offered to all participants.

Following the work of Campbell et al. [35] we assume that LS is directly influenced by satisfaction in different life domains (bottom-up). LS and satisfaction in six different life domains (living situation, financial situation, leisure time, health, family, neighbors and friends) was assessed using one question per domain “How satisfied are you with your [domain / overall in your life]?”, and the answer format was a 5-point Likert scale. The exact wording of the question and choice of life domains was taken from the German Cardiovascular Prevention Trial [27] and has been used in the German National Health Surveys for several decades [36]. Moreover, research has shown the reliability of single item measures of LS (e.g., [37, 38]).

The self-reported number of children was categorized into no children and any children without differentiating whether the children were adopted or biological. The children’s place of residence was asked, the answer categories were “In Bremen” and “Somewhere else” followed by an open text were respondents were asked to note down the places of residence. For respondents with more than one child multiple answers could be given. The variable was categorized into “Living in the same city” if at least one child was living in Bremen.

To assess frequency of contact with children the question “How often do you see your children?” was asked. For analyses, the response categories were recoded into number of meetings per year: “about once a week or more” into 104 meetings per year, “about once or twice a month” into 18 meetings per year, “several times a year” into 6 meetings per year, and “hardly ever” into 2 meetings per year, resulting in a continuous variable quantifying the meetings per year. To meet statistical assumption of the regression models, we log-transformed the variable.

Work in childcare was assessed by asking “What is your current situation? Do you have an unpaid or paid occupation?” Childcare was one of the answer categories, multiple answers were allowed.

Education was asked using two questions, one for school education and one for vocational training, which is highly formalized in Germany. Education was then classified according to the International Standard Classification of Education (ISCED) [39].

Additionally, Information of participant’s age (in years), sex (male, female), and family status (unmarried, married, divorced, widowed) was used in the present paper.

### Statistical analyses

Descriptive analyses with relative and absolute frequencies for sex, family status, children’s place of residence, frequency of contact with children, work in childcare as well as education were presented. Means and standard deviations were determined for age.

Linear regressions were performed to test for associations of LS with the different investigated life domains, frequency of contact with children, and work in childcare. Numerical LS scores were used for the regression models. All analyses were done for participants with and without children separately. Linear regressions were adjusted for sex and age. For each of the models, studentized residuals were calculated to check violations of regression model assumptions. All statistical analyses were performed with complete cases. The number of missing values is displayed in the (S1 Table).

All statistical analyses were conducted with SPSS 22.0 (IBM Corp. Armonk, NY). A p-value of <0.05 was considered statistically significant.

## Results

Characteristics of the study population are displayed in Table 1. 82.8% of the study population had at least one child. Of those, 51.2% were female and the mean age was 69.8±3.0 years. Among the participants without any children, 54.3% were female and the mean age was 69.4±2.8 years. Educational status was slightly higher among the participants without children (advanced education: 46.7% with children vs. 52.4% without children). More of the participants with children were married in comparison to the childless participants (70.9% vs. 48.7%). Of the participants with children, 59.5% had at least one of their children living in the same city. 49.5% saw one of their children at least once a week and 20.2% at least one or two times per month. More participants with children than participants without own children were involved in paid or unpaid childcare (7.0% vs. 1.8%). Of the participants with children, 43.2% indicated that they were very satisfied with their life in comparison to 37.5% of the childless participants.

**Table 1:** Description of the study sample.

	Total	With children (n = 1637)	Childless (n = 341)
	(N = 1978)		
	Mean (SD)	Mean (SD)	Mean (SD)
Age (in years)	69.8 (3.0)	69.8 (3.0)	69.4 (2.8)
	n (%)	n (%)	n (%)
Sex			
*Women*	1023 (51.7)	838 (51.2)	185 (54.3)
*Men*	955 (48.3)	799 (48.8)	156 (45.7)
Education			
*Basic education (ISCED level 1 + 2)*	241 (12.2)	204 (12.5)	37 (10.9)
*Specialized education (ISCED level 3 + 4)*	789 (40.1)	664 (40.7)	125 (36.8)
*Advanced education (ISCED level ≥ 5)*	940 (47.7)	762 (46.7)	178 (52.4)
Family status			
*Single*	147 (7.5)	41 (2.5)	106 (31.3)
*Married*	1312 (67.0)	1147 (70.9)	165 (48.7)
*Divorced*	277 (14.2)	240 (14.8)	37 (10.9)
*Widowed*	221 (11.3)	190 (11.7)	31 (9.1)
Children: place of residence			
*Living in the same city*	-	974 (59.5)	–
*Living somewhere else*	-	663 (40.5)	–
Children: frequency of contact			
*At least once a week*	-	763 (49.5)	–
*1–2 times per month*	-	311 (20.2)	–
*Several times per year*	-	414 (26.8)	–
*(Almost) never*	-	54 (3.5)	–
Paid or unpaid work in childcare			
*Yes*	119 (6.1)	113 (7.0)	6 (1.8)
*No*	1830 (93.9)	1499 (93.0)	331 (98.2)
Life satisfaction			
*5 very satisfied*	835 (42.2)	707 (43.2)	128 (37.5)
*4*	885 (44.7)	732 (44.7)	153 (44.9)
*3*	214 (11.0)	159 (9.7)	55 (16.1)
*2*	32 (1.6)	27 (1.6)	5 (1.5)
*1 very unsatisfied*	12 (0.6)	12 (0.7)	0

ISCED International Standard Classification of Education

Table 2 depicts the results for the six life domains. Most participants were very satisfied across all domains with the exception of satisfaction with health (22.0% very satisfied). Apart from satisfaction with family (very satisfied: 52.7% with children vs. 39.9% without children), the results are comparable for participants with children and childless participants.

**Table 2:** Description of the life domains.

	Total	With children (n = 1637)	Childless (n = 341)
	(N = 1978)		
Satisfaction with live domains	n (%)	n (%)	n (%)
Living situation			
*5 very satisfied*	1292 (65.3)	1077 (65.8)	215 (63.0)
*4*	502 (25.4)	412 (25.2)	90 (26.4)
*3*	128 (6.5)	98 (6.0)	30 (8.8)
*2*	31 (1.6)	28 (1.7)	3 (0.9)
*1 very unsatisfied*	25 (1.3)	22 (1.3)	3 (0.9)
Financial situation			
*5 very satisfied*	848 (42.9)	714 (43.6)	134 (39.3)
*4*	694 (35.1)	563 (34.4)	131 (38.4)
*3*	290 (14.7)	243 (14.8)	47 (13.8)
*2*	96 (4.9)	76 (4.6)	20 (5.9)
*1 very unsatisfied*	50 (2.5)	41 (2.5)	9 (2.6)
Leisure time			
*5 very satisfied*	1042 (52.7)	885 (54.1)	157 (46.0)
*4*	657 (33.2)	532 (32.5)	125 (36.7)
*3*	210 (10.6)	164 (10.0)	46 (13.5)
*2*	47 (2.4)	36 (2.2)	11 (3.2)
*1 very unsatisfied*	22 (1.1)	20 (1.2)	2 (0.6)
Health			
*5 very satisfied*	435 (22.0)	361 (22.1)	74 (21.7)
*4*	835 (42.2)	689 (42.1)	146 (42.8)
*3*	468 (23.7)	387 (23.6)	81 (23.8)
*2*	155 (7.8)	127 (7.8)	28 (8.2)
*1 very unsatisfied*	85 (4.3)	73 (4.5)	12 (3.5)
Family			
*5 very satisfied*	999 (50.5)	863 (52.7)	136 (39.9)
*4*	610 (30.8)	485 (29.6)	125 (36.7)
*3*	235 (11.9)	188 (11.5)	47 (13.8)
*2*	88 (4.4)	63 (3.8)	25 (7.3)
*1 very unsatisfied*	46 (2.3)	38 (2.3)	8 (2.3)
Neighbors and friends			
*5 very satisfied*	860 (43.5)	730 (44.6)	130 (38.1)
*4*	826 (41.8)	676 (41.3)	150 (44.0)
*3*	237 (12.0)	189 (11.5)	48 (14.1)
*2*	42 (2.1)	32 (2.0)	10 (2.9)
*1 very unsatisfied*	13 (0.7)	10 (0.6)	3 (0.9)

LS was statistically significantly related to all investigated life domains in both groups, independent of sex and age (see Table 3). For the participants with children, LS had the highest association with satisfaction with family (β: 0.202; 95%CI: 0.170–0.235), followed by satisfaction with neighbors and friends (β: 0.151; 95%CI: 0.111–0.191), and satisfaction with health (β: 0.148; 95%CI: 0.120–0.176). In comparison to that, childless participants had the highest association between LS and satisfaction with health (β: 0.193; 95%CI: 0.135–0.252), followed by satisfaction with family (β: 0.175; 95%CI: 0.114–0.236) and satisfaction with neighbors and friends (β: 0.154; 95%CI: 0.077–0.232). The correlation coefficients between the independent variables of the total model are displayed in the (S2 Table).

**Table 3:** Association of life satisfaction and satisfaction in life domains. Linear regression adjusted for sex and age.

	Life satisfaction (dependent variable)		
	Total	With children	Childless
	(N = 1978)	(n = 1637)	(n = 341)
	β	β	β
	(95%CI)	(95%CI)	(95%CI)
Satisfaction with life domains (independent variables)			
*Living situation*	0.115	0.112	0.132
	(0.080–0.150)***	(0.074–0.151)***	(0.046–0.217)**
*Financial situation*	0.115	0.116	0.103
	(0.088–0.142)***	(0.086–0.147)***	(0.041–0.165)**
*Leisure time*	0.141	0.141	0.146
	(0.106–0.175)***	(0.103–0.179)***	(0.065–0.226)***
*Health*	0.155	0.148	0.193
	(0.129–0.180)***	(0.120–0.176)***	(0.135–0.252)***
*Family*	0.199	0.202	0.175
	(0.170–0.228)***	(0.170–0.235)***	(0.114–0.236)***
*Neighbors and friends*	0.153	0.151	0.154
	(0.117–0.188)***	(0.111–0.191)***	(0.077–0.232)***
R^2^	0.52	0.51	0.57

** *p*-value < 0.01,

*** p-value < 0.001

Among participants with children, the satisfaction with family was positively associated with the frequency of contact to their children (β: 0.135; 95%CI: 0.099–0.171) (see Table 4).

**Table 4:** Association of satisfaction with family and frequency of contact in participants with children. Linear regression adjusted for sex and age.

	Family satisfaction (dependent variable)
	With children (n = 1542)
	Β
	(95%CI)
Children: frequency of contact	0.135
(number of meetings per year, log-transformed)	(0.099–0.171)***

*** p-value < 0.001

Table 5 depicts the association between LS and work in childcare for participants with and without children. In participants with children, there was a non-significant negative association between LS and work in childcare (β: -0.031; 95%CI: -0.178–0.116), while LS was statistically significantly positively associated to work in childcare in participants without own children (β: 0.681; 95%CI: 0.075–1.288).

**Table 5:** Association of life satisfaction and work in childcare. Linear regression adjusted for sex and age.

	Life satisfaction (dependent variable)	
	With children (n = 1612)	Childless (n = 337)
	β	β
	(95%CI)	(95%CI)
Paid or unpaid work in childcare	-0.031	0.681
	(-0.178–0.116)	(0.075–1.288)*

* *p*-value < 0.05

## Discussion

The goal of the present study was to investigate how satisfaction with seven life domains (i.e., living situation, financial situation, leisure time, health, family, as well as neighbors and friends) is related to overall LS in older adults. We furthermore examined influences of parental status and specifically investigated the differential role of intergenerational contact for LS among older adults with and without children.

We found that family as well as health satisfaction showed the strongest relationships with LS in the overall sample. This is in line with research highlighting the importance of social relationships for (older) adults’ well-being [10, 26] and findings demonstrating that health is a key contributor to subjective well-being [3, 15]. The findings of the present study furthermore provide additional evidence that the domain-specific approach to LS can elucidate differences in the correlates of LS and well-being between subpopulations. Past research has for instance shown that gender [40] and race [25] can affect relationships between domain-specific LS and overall LS. We extended these findings, showing that family satisfaction has the strongest association with LS in older adults with children, while health was most strongly related to LS among childless older adults. However, it is important to note that among childless older adults, family satisfaction and satisfaction with neighbors and friends had the second and third strongest relationships with LS, respectively. Similarly, following satisfaction with neighbors and friends, health had the third strongest association with LS among older adults with children. This suggests that for both groups, social relationships and health might be major contributors to overall LS. Among older adults with children, contact frequency with their children related positively with family satisfaction. This is consistent with previous research demonstrating that intergenerational contact frequency within the family is positively associated with well-being [31].

Interestingly, work with children was only significantly positively associated with LS among childless but unrelated to LS among older adults with children. Previous research has demonstrated the importance of intergenerational contact for older adults’ well-being [41] and has also shown that working in childcare can be very rewarding for older adults, who may feel needed and valued [42]. While our results should be interpreted with caution due to the small sample size of older adults who engage in paid or unpaid work with children, it seems as if working with children may be more beneficial for childless older adults’ LS. Research has shown that childless older adults have less contact with younger generations within the family than older adults with children [30]. Hence, childless older adults may compensate for fewer intergenerational family contacts by working with children. They perhaps also utilize their work with children more in order to express their generativity motives than older adults with children, who may express generativity through parenthood. Research has indeed shown that generativity is equally important to the well-being of parents and childless adults, yet childless adults may seek different activities to express their generativity motives [43], working with children possibly being one of them.

Older adults with children who work in childcare may predominantly provide care to their grandchildren and previous research has revealed mixed findings regarding potential benefits of grandparental childcare [44]. More specifically, the benefits of taking care of grandchildren appear to be contingent on the amount of caregiving and the strain associated with it. An important task for future research is thus to closer examine whose children childless older adults as well as older adults who are parents take care of (e.g., grandchildren, family members, and non-kin children) and how the type of caretaking affects LS.

### Limitations

The present study has some methodological limitations that should be addressed by future research. Our data was cross-sectional which precludes us from drawing conclusions regarding causal relationships. There has been a longstanding debate among researchers if LS is best predicted by satisfaction with various life domains (“bottom-up” perspective) or by stable traits, such as personality [26]. While we adopted a bottom-up perspective, assuming that satisfaction with life domains predicts LS, we cannot test this with the available data. We furthermore could not disentangle potential differences between effects of engaging in paid versus unpaid work in childcare on LS or elucidate the mechanisms through which it affects LS in childless older adults.

## Conclusions

Findings from this study add to the growing body of research that examines LS as a multi-dimensional construct and suggest that overall, satisfaction with social relationships as well as health are most strongly related to overall LS of older adults. The study also highlights differences in relations between domain-specific LS and overall LS between older adults who have children and older adults without children. Results further indicate that engaging in work with children, a form of intergenerational contact, may be more beneficial to childless older adults compared to older adults who have children. The results still warrant replication in other samples. Besides, future studies on this topic should employ longitudinal designs and use more fine-grained measures of intergenerational contact.

## Supporting information

S1 Table Number of missing values for all included variables. (DOCX)

S2 Table Spearman correlations between investigated life domains, sex and age. (DOCX)
