# Correction: Domain-specific life satisfaction among older adults with and without children: The role of intergenerational contact

**DOI:** 10.1371/journal.pone.0335483

**Published:** 2025-10-28

**Authors:** Friederike Doerwald, Birte Marie Albrecht, Imke Stalling, Karin Bammann

## Notice of republication

An incorrect version of the manuscript was published in error. This article was republished on September 30, 2025, to correct for this error. Please download this article again to view the correct version.
