# Supplementary material for: Domain-specific life satisfaction among older adults with and without children: The role of intergenerational contact
Source: PLoS One. 2021 Sep 22;16(9):e0257048. doi: 10.1371/journal.pone.0257048 (PMC8457449; doi:10.1371/journal.pone.0257048)
Supplement: S1 Table — (DOCX) [file pone.0257048.s001.docx]

**S1 Table.** Number of missing values for all included variables

|  | Total | With children | Childless |
| --- | --- | --- | --- |
| Age | 0 | 0 | 0 |
| Sex | 0 | 0 | 0 |
| Education | 8 | 7 | 1 |
| Family status | 21 | 19 | 2 |
| Children: place of residence | - | 0 | - |
| Children: frequency of contact | - | 95 | - |
| Paid or unpaid work in childcare | 29 | 25 | 4 |
| Life satisfaction | 0 | 0 | 0 |
| Satisfaction with life domains |  |  |  |
| *Living situation* | 0 | 0 | 0 |
| *Financial situation* | 0 | 0 | 0 |
| *Leisure time* | 0 | 0 | 0 |
| *Health* | 0 | 0 | 0 |
| *Family* | 0 | 0 | 0 |
| *Neighbours and friends* | 0 | 0 | 0 |
|  |  |  |  |
