# Supplementary material for: Domain-specific life satisfaction among older adults with and without children: The role of intergenerational contact
Source: PLoS One. 2021 Sep 22;16(9):e0257048. doi: 10.1371/journal.pone.0257048 (PMC8457449; doi:10.1371/journal.pone.0257048)
Supplement: S2 Table — (DOCX) [file pone.0257048.s002.docx]

**S2 Table.** Spearman correlations between investigated life domains, sex and age

|  | Satisfaction with life domains | | | | | |  |  |
| --- | --- | --- | --- | --- | --- | --- | --- | --- |
|  | *Living situation* | *Financial situation* | *Leisure time* | *Health* | *Family* | *Neighbours and friends* | Sex | Age |
| Satisfaction with life domains |  |  |  |  |  |  |  |  |
| *Living situation* |  | 0.38*** | 0.38*** | 0.27*** | 0.36*** | 0.37*** | 0.03 | 0.05* |
| *Financial situation* |  |  | 0.40*** | 0.30*** | 0.32*** | 0.28*** | 0.01 | 0.04 |
| *Leisure time* |  |  |  | 0.39*** | 0.38*** | 0.39*** | 0.01 | 0.04 |
| *Health* |  |  |  |  | 0.29*** | 0.28*** | -0.01 | 0.00 |
| *Family* |  |  |  |  |  | 0.48*** | -0.05* | 0.00 |
| *Neighbours and friends* |  |  |  |  |  |  | 0.08** | 0.03 |
| Sex |  |  |  |  |  |  |  | 0.01 |
| Age |  |  |  |  |  |  |  |  |
|  |  |  |  |  |  |  |  |  |

* p-value <0.05, ** p-value < 0.01, *** p-value < 0.001
